# The role of angiotensin-converting enzyme 2 (ACE2) receptor in the intestine in COVID-19: more research needed 

**Published:** 2020

**Authors:** Char Leung, Alice PY Wong

**Affiliations:** *Deakin University *

 Despite being a respiratory disease caused by SARS-CoV-2, a human betacoronavirus, COVID-19 can cause gastrointestinal symptoms such as diarrhea and nausea. While the etiology and pathogenesis of COVID-19 remain not clearly known, it has been suggested that angiotensin-converting enzyme 2 (ACE2), a protein mediating the cell entry of SAR-CoV and SAR-CoV-2, might play a role in the transmission given its surface expression on lung alveolar epithelial cells as well as enterocytes of the small intestine (1). Therefore, a number of studies have purposed to investigate the link between ACE2 and the gastrointestinal symptoms caused by COVID-19 (2,3). However, a number of issues and much debate remain unresolved and, hence, require further research. 

Can ACE2 explain the gender differences in susceptibility? A number of clinical studies in China have observed a higher prevalence of COVID-19 in males (4,5). Thus, it has been proposed that ACE2 plays a role as it is highly expressed in Asian men (6). However, ACE2 is also the receptor for SARS-CoV, and a higher prevalence of SARS in females has been observed (7-10). The following questions remain unresolved. Is ACE2 equally expressed in the intestine in males and females? If so, why does it fail to explain the gender differences in susceptibility? Does the gender ratio disparity affect the observed higher prevalence in males in China? Do clinical findings in Caucasians also demonstrate a higher prevalence in males? Other than ACE2, is any other protein involved in the host cell entry in COVID-19 but not in SARS, although SARS-CoV and SARS-CoV-2 are genetically similar?

Can ACE2 explain the link between the gastrointestinal symptoms observed in a patient and how the patient is infected? The answer to this question is of great importance to infection control, as it helps identify the source of infection by identifying the route of transmission. In an outbreak of SARS-CoV in a residential building in Hong Kong where the faulty sewer system was believed to have spread the virus, the virus RNA was detected in 97% of the patients’ stool samples, and 73% of all patients developed diarrhea (10). If the expression of ACE2 in the intestine is proven to contribute to gastrointestinal symptoms, the mode of transmission of SARS-CoV-2 can be further clarified.

To conclude, the gastroenterological aspects of COVID-19 remain an unexplored area. While many studies have delivered important clinical findings for the control and management of COVID-19, little is known about the pathophysiology of diarrhea, vomiting, and nausea in COVID-19 patients. More research should be done, and the role of ACE2 may serve as a hint. 
